# Somatostatin Serves a Modulatory Role in the Mouse Olfactory Bulb: Neuroanatomical and Behavioral Evidence

**DOI:** 10.3389/fnbeh.2019.00061

**Published:** 2019-04-09

**Authors:** Sonia Nocera, Axelle Simon, Oriane Fiquet, Ying Chen, Jean Gascuel, Frédérique Datiche, Nanette Schneider, Jacques Epelbaum, Cécile Viollet

**Affiliations:** ^1^INSERM, UMR 894-Center for Psychiatry and Neuroscience (CPN), Paris, France; ^2^Université Paris Descartes-Sorbonne Paris Cité, Paris, France; ^3^CNRS UMR 6265—Centre des Sciences du Goût et de l’Alimentation (CSGA), Dijon, France

**Keywords:** somatostatin receptor, mitral cells, interneurons, olfaction, SSTR3, SSTR2, SSTR4, knockout mice

## Abstract

Somatostatin (SOM) and somatostatin receptors (SSTR1–4) are present in all olfactory structures, including the olfactory bulb (OB), where SOM modulates physiological gamma rhythms and olfactory discrimination responses. In this work, histological, viral tracing and transgenic approaches were used to characterize SOM cellular targets in the murine OB. We demonstrate that SOM targets all levels of mitral dendritic processes in the OB with somatostatin receptor 2 (SSTR2) detected in the dendrites of previously uncharacterized mitral-like cells. We show that inhibitory interneurons of the glomerular layer (GL) express SSTR4 while SSTR3 is confined to the granule cell layer (GCL). Furthermore, SOM cells in the OB receive synaptic inputs from olfactory cortical afferents. Behavioral studies demonstrate that genetic deletion of SSTR4, SSTR2 or SOM differentially affects olfactory performance. SOM or SSTR4 deletion have no major effect on olfactory behavioral performances while SSTR2 deletion impacts olfactory detection and discrimination behaviors. Altogether, these results describe novel anatomical and behavioral contributions of SOM, SSTR2 and SSTR4 receptors in olfactory processing.

## Introduction

The neuropeptide somatostatin (SOM, encoded by the *sst* gene) is found in most regions of the central nervous system. It is expressed both in local interneurons and long-projecting neurons that connect distant brain regions. SOM is known to exert neuromodulatory actions on cognitive, emotional and sensory behaviors through the activation of specific receptors (SSTR1–4 in the central nervous system, SSTR5 in the periphery; Martel et al., 2012; Liguz-Lecznar et al., 2016). SOM receptors are localized in dendritic domains of principal cells or interneurons in most brain regions where they contribute to the fine-tuning of neuronal activity, shaping synaptic activity and plasticity of the central nervous system (Large et al., 2016). In addition to the large set of literature related to the cortical and hippocampal distribution of SOM neuronal networks, several studies have described SOM distribution in human and rodent olfactory processing pathways (Videau et al., 2003; Lepousez et al., 2010a; Brunjes et al., 2011; Martel et al., 2012; De La Rosa-Prieto et al., 2016; Large et al., 2016; Saiz-Sanchez et al., 2016). The recent description of different combinations of SSTR subtypes in each olfactory structure from the bulb to the olfactory cortex (Martel et al., 2015) suggests that the SOM peptide modulates different stages of olfactory processing. Pharmacological and behavioral data support this hypothesis, showing that activation or blockade of somatostatin receptor 2 (SSTR2) transduction in the murine olfactory bulb (OB) respectively increases or decreases olfactory fine discrimination as well as basal gamma oscillations in the OB (Lepousez et al., 2010b).

The OB is the first telencephalic relay in olfactory processing and it shows a typical cytoarchitecture: each concentric bulbar layer contains distinct interneuron cell populations with specific neurochemical and anatomical features that participate in the local synaptic shaping of the olfactory signal driven by the mitral and tufted cells, the principal neurons of the OB (for review, see Nagayama et al., 2014). Apical primary dendrites of the principal cells receive afferent synaptic inputs from olfactory neurons that project from the olfactory epithelium into the peripheral glomerular layer (GL). Their long axonal projections relay the signal to the anterior olfactory nucleus (AON) and to downstream structures of the olfactory cortex, i.e., olfactory tubercle for tufted cells, piriform cortex and entorhinal cortex and limbic regions for mitral cells. In between, major synaptic interactions take place in each OB layer. In the GL, intrinsic inhibitory circuits control both gain and strength of the sensory inputs in a spatial and temporal manner (Wilson et al., 2014; Linster and Cleland, 2016; Chong and Rinberg, 2018). In the external plexiform layer (EPL), dendro-dendritic reciprocal synapses occur between lateral dendrites of the mitral cells and dendrites of granule cell interneurons, the main inhibitory population of the OB, whose somata are located in deepest layer, the granule cell layer (GCL). These reciprocal synaptic interactions induce local field potential oscillations, including gamma oscillations which are involved in fast discrimination between close stimuli in the OB (Frederick et al., 2016) and feed-forward transmission of the signal to downstream associational regions of the olfactory cortex (Kay, 2014). Finally, retrograde afferents from the olfactory cortex and other brain structures also target the OB (Shipley and Ennis, 1996; Lepousez and Lledo, 2013; Kay, 2014; Wilson et al., 2014; Diodato et al., 2016; Case et al., 2017), and modify local synaptic activity (Balu et al., 2007; Boyd et al., 2012; Devore and Linster, 2012; Markopoulos et al., 2012; Soria-Gómez et al., 2014; Brunert et al., 2016; Sanz Diez et al., 2017).

In mouse OB, SOM is predominantly expressed in calretinin-expressing GABAergic interneurons of the inner EPL and in sparse GABAergic deep short-axon cells and fibers in the GCL (Lepousez et al., 2010a). It is hardly detected in the GL (Lepousez et al., 2010a; De La Rosa-Prieto et al., 2016; Burton et al., 2017). Recent anatomical and physiological data revealed that EPL interneurons, including those expressing SOM, interact with mitral cell dendrites via dendro-dendritic reciprocal synapses (Hamilton et al., 2005; Lepousez et al., 2010a; Huang et al., 2013), similar to granule cells. This anatomically supports the tonic regulation by endogenous SOM of basal gamma oscillations in the OB and olfactory behavior (Lepousez et al., 2010b).

Besides the peptide itself, SOM receptors are strongly expressed in the OB (Videau et al., 2003; Martel et al., 2015). The present study was undertaken to: (i) precisely identify their cellular localization using reliable immunohistochemical tools and transgenic models; (ii) determine the neural afferents targeting OB SOM neurons using viral tracing; and (iii) study how genetically impairing SOM transduction impacts olfactory performance. Our results show that SSTR4 and SSTR3 receptors are expressed in distinct inhibitory interneuronal populations, respectively located in GL and GCL. SSTR2 receptors are clearly expressed in a subpopulation of mitral cell-like neurons. Furthermore, we show that local SOM-expressing neurons receive feedback projections from downstream regulatory regions of the olfactory cortex. This indicates that endogenous and centrifugal SOM can specifically target all the key dendritic regulation sites of the olfactory mitral cell-mediated transmission in the OB. Genetic ablation of SOM, SSTR2 or SSTR4 show limited effects on olfactory behavioral performances, with no major impact on olfactory learning or memory. Olfactory detection and discrimination performances are impaired in SSTR2 KO mice as compared to WT but SSTR4 KO and SOM KO do not show such changes. These differential results suggest a multimodal somatostatinergic control of olfactory processing, pointing to different cellular and behavioral contributions of each SSTR subtype.

## Materials and Methods

### Animals

All procedures were approved by a local ethics committee (French Ministry of Health and Research Authorization N° 00618.04 and APAFIS#5670-2016120716328268) in accordance with the European Communities Council Directive (86/609/EU) and the European Union guidelines. Mice were bred and housed in the CPN animal facility on a 12 h light/dark cycle with *ad libitum* access to food and water except during behavioral experiments. Control (WT) and transgenic littermates from constitutive knock-out (KO) transgenic lines, *sst* KO (referred as SOM KO in the text; Low et al., 2001), *sstr2* KO (SSTR2 KO, Viollet et al., 2000) and *sstr4* KO (SSTR4 KO, Helyes et al., 2009) as well as SSTR2 KO-LacZ KI (Allen et al., 2003) and Kv3.1-EYFP animals (Metzger et al., 2002) were used for immunohistochemical studies. Five to 8-week-old SOM-IRES-Cre heterozygous males (Taniguchi et al., 2011) were used for viral studies. Three independent cohorts of eight age-matched transgenic and control (WT) male mice (3–5 months) from SOM, SSTR2 or SSTR4 KO transgenic lines were used for the behavioral sequences. Animals which did not perform all operant tasks were excluded from statistical analysis (1 WT and 1 SOM KO mice, 1 SSTR2 KO mouse, 1 WT and 1 SSTR4 KO mice). Experimenters were blind to the genotype of the animals during both experiments and analysis.

### Immunohistochemistry

#### Mouse Samples

Mice (at least three per group) were deeply anesthetized with an intraperitoneal injection of ketamine/xylazine mixture (100 mg/kg/7 mg/kg in saline) and then transcardially perfused with 4% paraformaldehyde (PFA) in 0.1 M phosphate buffer pH 7.4. Brains were quickly removed, post-fixed for 2 h in 4% PFA, cryoprotected (30% sucrose in PBS), fast-frozen at −40°C in isopentane and sectioned in 40 μm coronal sections using a microtome (Leica). After several washing steps in Tris buffer saline (TBS), sections were incubated for 30 min at room temperature in the blocking solution (10% normal donkey serum (NDS), 0.3% Triton X100 in TBS) then primary antibodies were incubated for 24 h (4 days for Arl13b staining; see Table 1) in 2% NDS, 0.3% Triton X100 in TBS. After three washes in TBS, sections were incubated for 2 h with appropriate Alexa488-, Cy3- and Cy5-conjugated donkey secondary antibodies (Jackson ImmunoResearch, respective dilutions: 1/500, 1/1,000, 1/200) in the same buffer. After three TBS rinses, sections were mounted beneath coverslips with Fluoromount G mounting medium onto glass slides (Southern Biolabs). For Arl13b, reelin or GAD67, sections were incubated 1 h in “Mouse On Mouse” solution (Vector Labs) before the blocking step. For GAD67 staining, sections were incubated during the whole procedure in Triton-free buffer including 0.1% sodium azide and with the primary antibodies for 7 days.

**Table 1:** Primary antibodies used in this study.

Primary antibody	Species (mono, polyclonal)	Dilution use	Source/Reference
Arl13b	Mouse (monoclonal)	1:500	#73-287, Antibodies Inc
Beta-Galactosidase (β-Gal)	Chicken (polyclonal)	1:2,500	# ab9361, Abcam
Calretinin (CR)	Goat (polyclonal)	1:4,000	#AB1550, Chemicon
GAD67	Mouse (monoclonal)	1:400	#MAB5406, Millipore
Green fluorescent protein (GFP)	Chicken (polyclonal)	1:1,000	#Ab 13970, Abcam
Neuronal nitric oxide synthase (nNOS)	Rabbit (polyclonal)	1:800	#61-7000, Invitrogen
Olfactory marker protein (OMP)	Goat (polyclonal)	1:20,000	# 544-10001, Wako
Parvalbumin (PV)	Mouse (monoclonal)	1:500	#P3088, Sigma
Reelin	Mouse (monoclonal)	1:500	#MAB5364, Millipore
Somatostatin	Goat (polyclonal)	1:500	#D20, sc-7819 Santa Cruz
Somatostatin receptor 2 (SSTR2)	Rabbit (monoclonal)	1:2,000	#ab134152, Abcam
Somatostatin receptor 3 (SSTR3)	Rabbit (polyclonal)	1:2,000	#PA3-207, Thermo Scientific

Sections were analyzed under a confocal laser scanning microscope (TCS SP5, Leica) under a 40× oil-immersion objective. Images were sequentially acquired for A488 and Cy5 or Cy3 fluorescent signals using single excitation beams (Ar laser at 488 nm wavelength, laser diode at 561 nm and HeNe laser at 633 nm). Displayed images correspond to 1.64–24.8 μm-thick stacks along the z-axis (0.4 μm step). Enlarged illustrations (Figures 2E,F, 3) correspond to 0.4 and 0.51 μm-thick stacks, respectively.

For cell density measurements, fluorescent immunostaining for SOM and SSTR2 performed on WT serial 40 μm coronal OB sections was imaged at constant light settings using a Lamina slide scanner (Perkin Elmer, ×20 objective) equipped with GFP and Cy3 filter sets. Each two-channel image was extracted using Pannoramic Viewer software and exported into Image J using Bio-Format importer plug-in. Six anterior anatomic levels (every 250 μm from Bregma 4.25) were analyzed with Image J with a dedicated macro transforming SSTR2 and SOM staining into masks in order to count the labeled cells or area in each region of interest (GL, EPL and GCL). Data were averaged from three to four mice per level.

### Viral Tracing

Polytrans-synaptic tracing was performed using PRVBa2001 virus, an attenuated Cre-dependent pseudorabies virus. This virus encodes a green fluorescent protein marker and replicates only in neurons that express cre recombinase and their presynaptic neurons, allowing the identification of neural inputs in a retrograde manner (DeFalco et al., 2001). PRVBa2001 solution titer was 3.10^9^ PFU/ml of culture media. Trace of fluorospheres (1 μm diameter, blue 365/415; 1:100 solution in 0.9% NaCl, Molecular Probes) was co-injected with PRVBa2001 (1/10 ratio) to localize the injection site.

Mice were injected in a stereotaxic frame using validated procedures under isofluorane gaseous anesthesia. A hole was drilled in the skull above the medial OB (Bregma 4 A-P axis and 0.8 M-L) to insert a 34-gauge blunt needle (World Precision Instruments) 1.7 mm deep. One-hundred nanoliter of the injection solution (PRVBA2001 virus + fluorospheres) was injected at 20 nl/min and left in place for 15 min to ensure proper injection and diffusion. Injection sites were checked *a posteriori* using detection of fluorospheres.

Mice were sacrificed 3–5 days post-injection in order to trace PRVBa2001 virus progression. Brains were then fixed as previously described and 2D reconstructions of serial anteroposterior coronal sections (50 μm thick, every 300 μm) were prepared and analyzed using Zeiss Axovision software on a Zeiss Imager M2 epifluorescence microscope (10× magnification). The neuroanatomical location of the positive labeled cells was determined using *the mouse brain in stereotaxic coordinates atlas* (Paxinos and Franklin, 2008).

### Behavior

Three separate sets of experiments were undertaken in order to determine the contribution of constitutively SOM, SSTR2 and SSTR4 knockout on olfaction. The details of the behavioral procedures are described in Table 2 (top). Each test was performed with a cohort of transgenic and WT littermate animals in a well-ventilated room by an experimenter blind to genotype. To reduce the duration of the water-restriction period, there was no interval between the tests.

**Table 2:** Details of the sequence of behavioral tests.

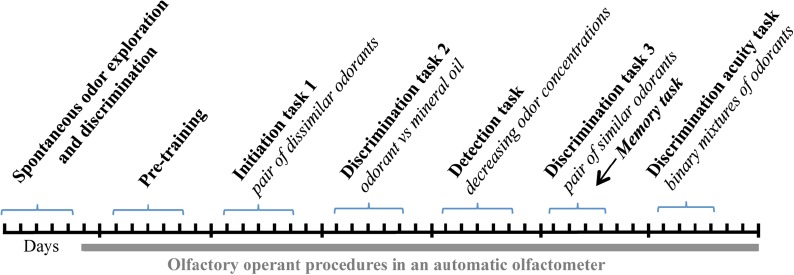					
		SOM KO	SSTR2 KO	SSTR4 KO
**Habituation/dishabituation task**			H: 1% pentanal, C+1: 1% hexanal, C+3: 1% octanal	
**Operant procedures**	Initiation task		S+: 1% anisole S−: 1% cineole	S+: 1% hexanal S−: 1% heptanal
	Discrimination task 2		S+: 1% isoamylacetate S−: mineral oil	S+: 1% (+) carvone S−: mineral oil
	Detection task		S+: isoamylacetate dilutions S−: mineral oil	S+: (+) carvone dilutions S−: mineral Oil
	Discrimination task 3		S+: 1% (+) carvone S−: 1% (−) carvone	
	Discrimination acuity task		S+: (+) carvone/(−) carvone binary mixtures S−: (+) carvone/(−) carvone inverse binary mixtures	
	Memory task (21d)		S+: 1% anisole S−: 1% cineole	S+: 1% hexanal S−: 1% heptanal

#### Spontaneous Odor Exploration and Discrimination in a Habituation-Dishabituation Protocol

Mice were tested in custom-built open plexiglass boxes (25 × 40 × 15 cm) made so that odorant stimuli (10 μl centered on a 5 cm diameter filter paper, Whatman) could be inserted at various places beneath a grid floor. Three different odorants (Sigma-Aldrich, 1% vol/vol in mineral oil) were used: the habituation odor pentanal (H) and two test odors of variable similarity, hexanal with one additional carbon chain (C+1) and octanal with three additional carbon chains (C+3). One week before the experiment, mice were housed individually and handled daily. Two days prior to the experiment, animals were habituated to the testing box for 20 min. Except for SOM cohort, mice were water-deprived the night before the test in order to increase motivation. The day of the experiment, mice were tested successively with freshly prepared odors: mice explored the box for 5 min before the habituation odor (H) was presented four consecutive times (H_1_ to H_4_ trials, 2 min each). Mice were then exposed for 2 min successively to C+1 test odor, again to the habituation odor (H_5_) and finally to C+3 test odor. Each odor presentation was followed by a 5 min inter-trial interval and the box was cleaned with water and alcohol between each session. Odor exploration, i.e., the time spent investigating the filter area, was recorded offline by an experimenter blind to the genotype of the mice. H_1_ to H_4_ data were analyzed to test the formation of a memory and habituation. Comparison between H and test odor trials (H4 vs. C_+1_, H5 vs. C_+3_) tested the ability to discriminate between the habituation odor and the test odor.

#### Olfactory Operant Conditioning

Mice were trained using custom-built computer-controlled four-channel olfactometers as previously described (Martel et al., 2015). Odorants (Sigma-Aldrich) were prepared daily and diluted vol/vol with odorless mineral oil (Sigma-Aldrich). Odors were generated by bubbling charcoal-filtered air in 10 ml of odorant in a 40 ml glass tube. The odorant vapor was mixed with clean air before its introduction into the sampling port (ratio 1:20).

Mice were first trained to the go/no-go procedure during five pre-training sessions to learn the operant procedure (for details, see Martel et al., 2015). Then mice were trained to respond to the presence of an odor (positive stimulus, S+) by licking the water port and to refrain from responding to the presence of another odor (negative stimulus, S−; Figure 10A). In each trial, a single stimulus (positive or negative) was presented. If the response criterion was met in S+ trials, a droplet of water (3 μl) was given as a reward and the trial was scored as a hit. Failing to lick in S− trial was scored as a correct rejection. S+ and S− trials were presented in a pseudo-random order, each block contained 10 S+ and 10 S− trials, never presented more than three times consecutively. The percentage of correct responses was determined for each block of 20 trials ((hits + correct rejections)/20 × 100) and scored for the 10 consecutive blocks of each session. Scores ≥ 85% implied that mice had correctly learned to assign the reward value of the S+ and the non-reward value of the S−. The number of blocks necessary to reach the 85% learning criterion (Blocks to criterion or BTC) was used to compare individual learning per group. To calculate BTCs, mice which did not reach the criterion were arbitrarily assigned one extra block. The three last blocks of the learning task were averaged to score the final performance level reached for each group.

**Figure 1:**
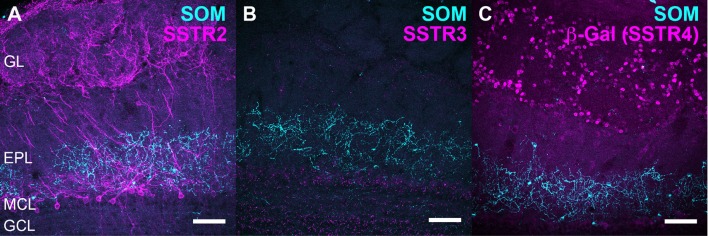
Relative distribution of somatostatin (SOM) and somatostatin receptor 2 (SSTR2), SSTR3 and SSTR4 receptors in the olfactory bulb (OB). **(A–C)** Double labeling of SOM (cyan) with SSTR2 **(A)**, SSTR3 **(B)** or SSTR4 (**C**, evidenced by nuclear β-Galactosidase immunoreactivity), (SSTR2 KO and SSTR2 magenta) in the mouse OB. Brain sections from wild-type **(A,B)** or SSTR4 KO-lacZ KI heterozygous **(C)** mice were used. **(A–C)** 45, 52, 35 confocal planes (0.41 μm) stacked for each illustration. EPL, external plexiform layer; GCL, granule cell layer; GL, glomerular layer; MCL, mitral cell layer; SSTR, somatostatin receptor. Scale bars: 50 μm.

**Figure 2:**
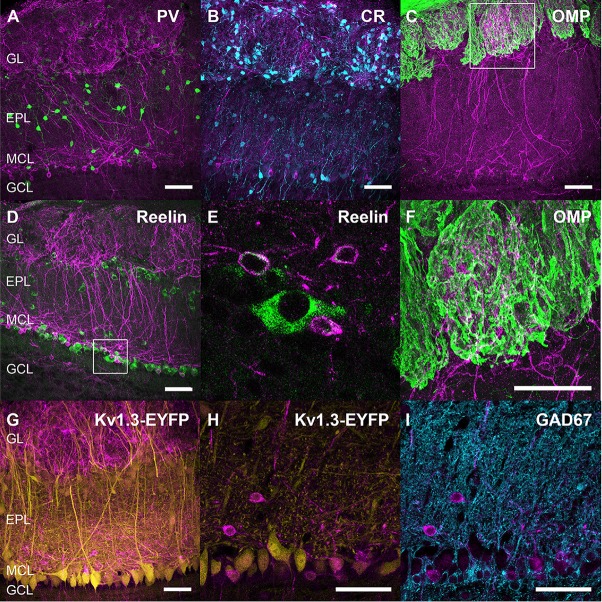
Neurochemical characterization of SSTR2-expressing neurons. Double labeling of SSTR2 (magenta) with parvalbumin (PV, green, **A**), calretinin (CR, cyan, **B**), olfactory marker protein (OMP, green, **C**, zoomed on a single 0.4 μm confocal plane in **F**), reelin (green, **D**, zoomed on two confocal planes in **E**) or GAD67 (cyan, **I**). Brain sections from wild-type **(A–F,I)** and Kv3.1-EYFP **(G,H)** heterozygous mice were used. Respective number of confocal planes stacked for each illustration: **(A–D,G)** 43, 60, 46, 45, 60, **(H,I)** 0.41 μm (3×0.17 μm). EPL, external plexiform layer; GCL, granule cell layer; GL, glomerular layer; MCL, mitral cell layer; SSTR, somatostatin receptor. Scale bars: 50 μm.

**Figure 3:**
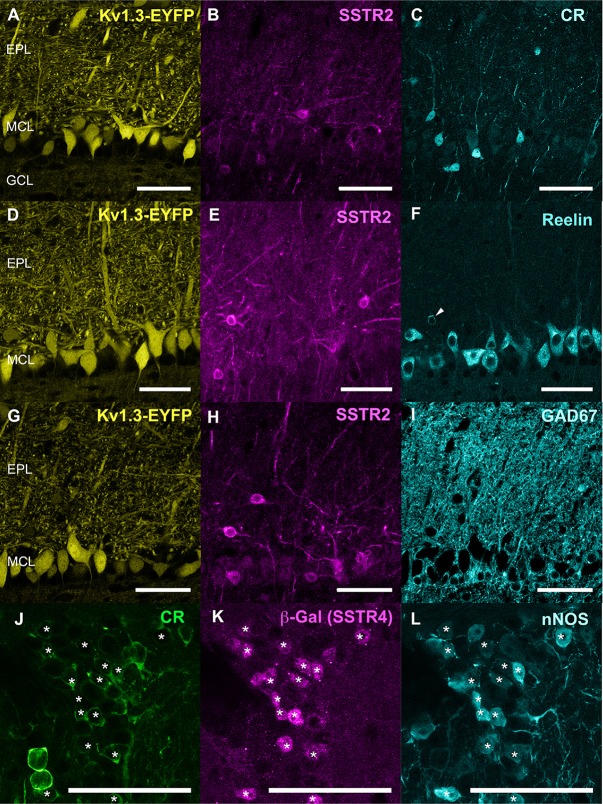
**(A–I)** Respective distribution of Kv3.1-EYFP, SSTR2 and CR **(A–C)**, Kv3.1-EYFP, SSTR2 and reelin **(D–F)**, Kv3.1-EYFP, SSTR2 and GAD67 **(G–I)**. Three confocal planes (0.4 μm step) were stacked for each illustration. **(J–L)** Colocalization (star) of calretinin (CR, green), b (write with a “beta” like in Figure 1)-Galactosidase (magenta) and nNOS (cyan) in the GL of SSTR4 KO-LacZ KI heterozygous mice (**J–L** on two stacked 0.4 μm confocal plane). Scale bars: 50 μm.

**Figure 4:**
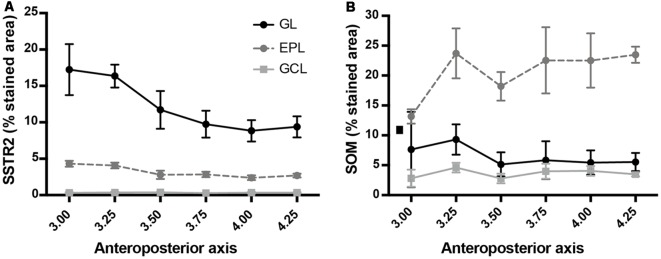
SSTR2 **(A)** and SOM **(B)** cell density along the anteroposterior axis of the main OB. Horizontal scale: Bregma levels.

**Figure 5:**
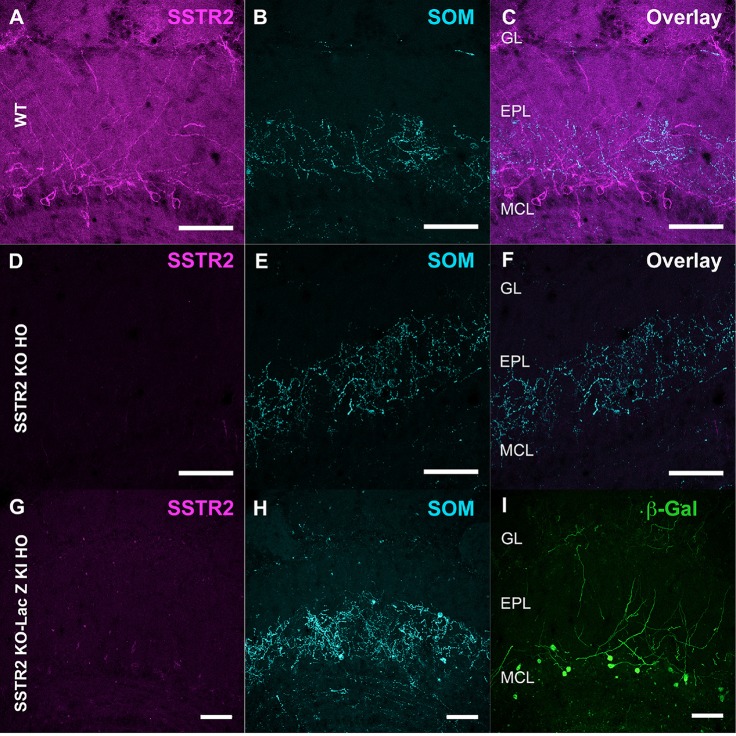
Specificity of SSTR2 labeling. **(A–F)** Immunolabeling of SSTR2 (**A**, magenta) and SOM (**B**, cyan) in WT **(A,B)** and homozygous SSTR2 KO mice **(D,E)**. Panels **(C,F)** show overlay (47 confocal planes). **(G–I)** Immunolabeling of SSTR2 (**G**, magenta), SOM (**H**, cyan) and β-Galactosidase (**I**, green) in homozygous SSTR2 KO-LacZ KI mice (67 confocal planes). Scale bars: 50 μm.

**Figure 6:**
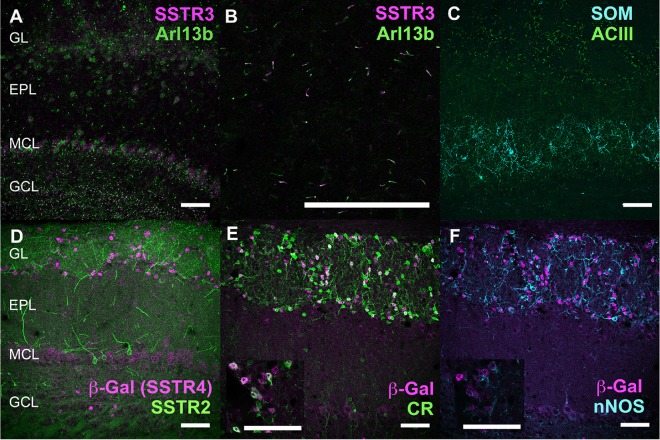
Neurochemical mapping of SSTR3 and SSTR4 in the OB. **(A–C)** Double labeling of SSTR3 (magenta) with Arl13b (green) primary cilia marker in the OB. **(B)** Higher magnification of Arl13b labeling superimposed to all SSTR3-labeled primary cilia in the IPL (arrows). **(C)** Double labeling of SOM (cyan) with Adenylyl cyclase 3 (ACIII, green, **C**). **(D–F)** Double-labeling of β-Galactosidase (magenta, visualizing SSTR4 expression) with SSTR2 (green, **D**), calretinin (CR, green, **E**), or neuronal nitric oxide synthase (nNOS, cyan, **F**) in the OB. Embedding of nuclear β-Gal signals in CR-labeled cells **(E)** and nNOS-labeled cells **(F)** is shown on a single 0.4 μm confocal plane in the respective inserts. Brain sections from wild-type **(A–C)** or SSTR4 KO-lacZ KI **(D–F)** mice were used. Respective number of planes stacked for each illustration **(A,C−F)**: 20, 40, 31, 15, 15 (0.4 μm). EPL, external plexiform layer; GCL, granule cell layer; GL, glomerular layer; MCL, mitral cell layer; RMS, rostral migratory stream. Scale bars: 50 μm.

**Figure 7:**
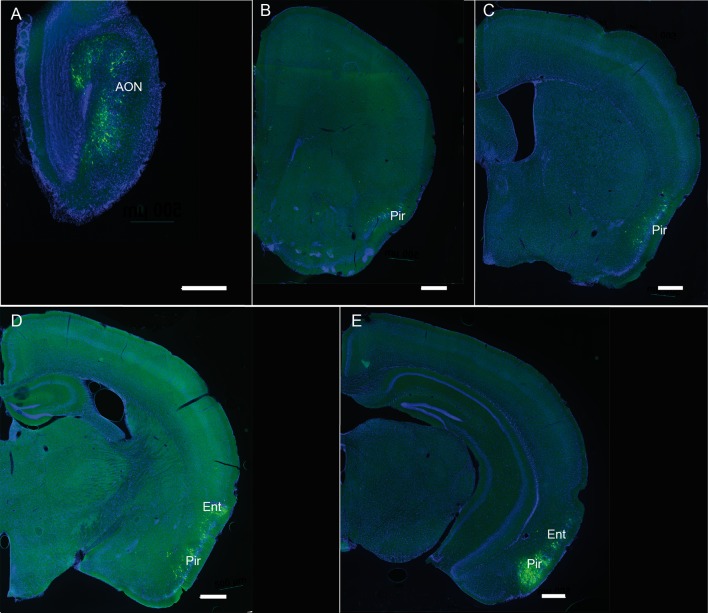
**(A–E)** Representative pictures showing the retrograde labeling observed ipsilaterally 3 days after injection of PRV Ba2001 Cre-dependent pseudorabies virus in the OB of heterozygous SOM-IRES-Cre mice. The following Bregma levels are illustrated: 3.17 **(A)**, 1.41 **(B)**, −0.11 **(C)**, −2.03 **(D)**, −2.79 **(E)**. AON, anterior olfactory nucleus; Pir, piriform cortex; Ent, entorhinal cortex; Green: GFP; blue: DAPI. Scale bars: 500 μm.

**Figure 8:**
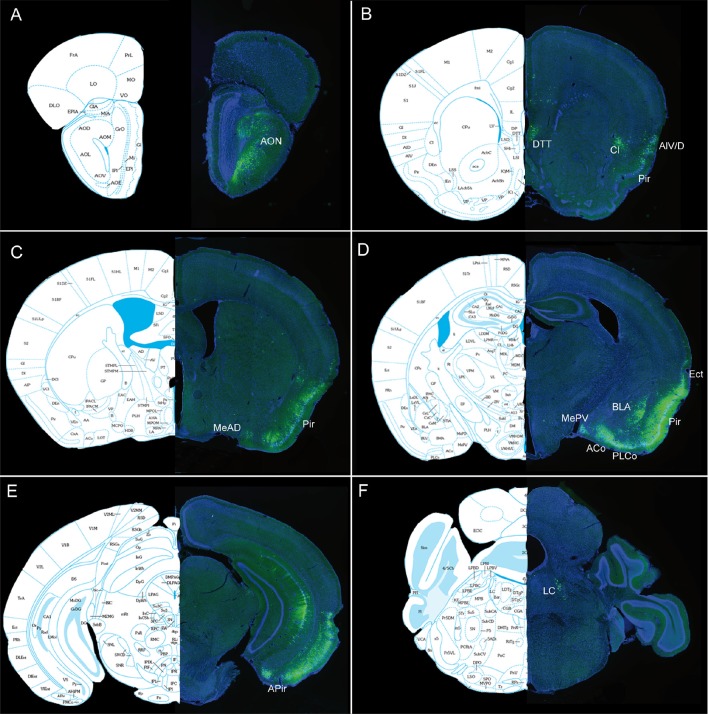
**(A–F)** Representative pictures showing PRVBa2001-infected neurons expressing GFP ipsilaterally 3 days after injection in the OB of heterozygous SOM-IRES-Cre mice. The following Bregma levels are illustrated: 3.56 **(A)**, 1.94 **(B)**, 0.02 **(C)**, −1.36 **(D)**, −3.51 **(E)**, -5.34 **(F)**. Aco Anterior cortical nucleus of amygdala, AIV/D agranular insular area, AON: anterior olfactory nucleus, BLA basolateral amygdala, APir : anterior piriform cortex, Cl claustrum, DTT dorsal tenia tecta, Ect ectorhinal cortex, Ent entorhinal cortex, MeAD medial nucleus of amygdala, MePV medial amygdaloid nucleus, Pir piriform cortex, LC locus coeruleus, PLCo posterolateral cortical amygdaloid area. Atlas pictures are based on Paxinos and Franklin (2008).

**Figure 9:**
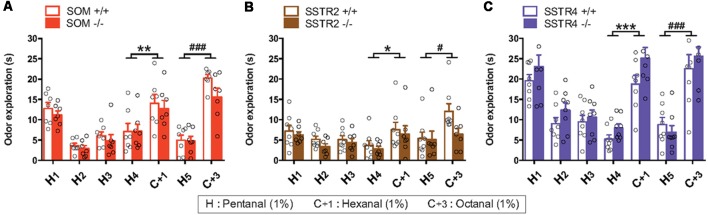
Spontaneous olfactory discrimination for SOM **(A)**, SSTR2 **(B)** and SSTR4 **(C)** transgenic mice cohorts using a habituation/dishabituation task. H, hexanal; C_+1_, heptanal; C_+3_, octanal. WT mice: empty bars, KO mice: plain bars. *n* = 7–8 mice per group. Inter-Trial Interval: 5 min. Open circles indicate individual values, error bars indicate SEM. Odor exploration **(A–C)**, trial effect: H_4_ vs. C_+1_ *P < 0.05, **P < 0.01, ***P < 0.001, H_5_ vs. C_+3_^#^*P* < 0.05, ^###^*P* < 0.001, two-way r-m ANOVA.

**Figure 10:**
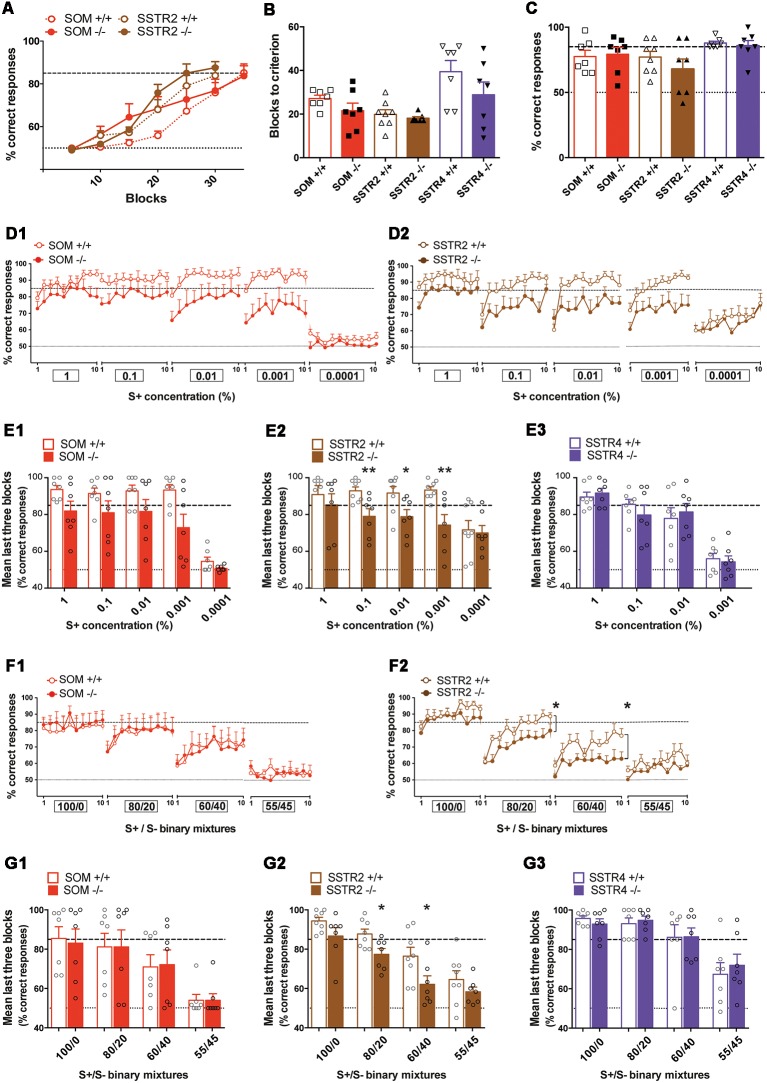
Olfactory performances of SOM KO and SSTR2 KO mice cohorts evaluated using operant tasks. **(A)** Learning curves for SOM and SSTR2 cohorts (Initiation task, odor pair: anisole/cineole). **(B)** Mean blocks to criterion (BTC) number for WT and KO groups in the initiation task. **(C)** Olfactory memory performances 21 days after completion of the initiation task. **(B,C)** Symbols show individual values. **(D)** Mean percentage of correct responses for each training session in the olfactory detection task. **(D_1_)** SOM cohort (*n* = 7), **(D_2_)** SSTR2 cohort (*n* = 7–8). Ten blocks per session, boxes indicate S^+^ concentrations (%). **(E)** Mean performance of the last three blocks in the olfactory detection task. **(E_1_)** SOM cohort (*n* = 7), **(E_2_)** SSTR2 cohort (*n* = 7–8), **(E_3_)** SSTR4 cohort (*n* = 7). Open circles indicate individual values. *P < 0.05, **P < 0.01 vs. WT, Bonferroni-corrected ANOVA. **(F)** Mean percentage of correct responses for each training session in the olfactory discrimination task. **(F_1_)** SOM cohort (*n* = 7), **(F_2_)** SSTR2 cohort (*n* = 7–8). Ten blocks per session, boxes indicate S+/S− mixtures. Group: *P < 0.05, r-m ANOVA. **(G)** Mean performance of the last three blocks in the discrimination task. **(G_1_)** SOM cohort (*n* = 7), **(G_2_)** SSTR2 cohort (*n* = 7–8), **(G_3_)** SSTR4 cohort (*n* = 7). *Open circles indicate individual values. *P < 0.05 vs. WT, Bonferroni-corrected ANOVA. Red: SOM cohort, Brown: SSTR2 cohort, Purple: SSTR4 cohort. WT mice: open symbols/bars, KO mice: filled symbols/bars. Black dashed line: 85% success criterion, gray dashed line: 50% chance level. Error bars indicate SEM.

Mice were submitted to an initiation task where they had to learn the rule and discriminate between dissimilar odorants (Table 2). This initial task is difficult for the mice and required between 30 and 35 blocks with anisole/cineole odor pair and 50 blocks with hexanal/heptanal odor pair to increase performances. The longer training with hexanal/heptanal is probably due to the close similarity of those latter chemicals, which increased the complexity of the task for the animals. This odor pair of the initiation task was later changed to anisole/cineole pair for SOM and SSTR2 experiments. One SSTR2 WT mouse was trained for 30 blocks instead of 35 and will not appear on Figure 10B, even if it reached the 85% criterion (*n* = 6 for this graph only).

Mice were then trained to discriminate between a novel odorant and odorless mineral oil (Discrimination Task 2). Once the 85% criterion was reached, they were tested for detection thresholds using decreasing concentrations of the odorant diluted in mineral oil as S+ (one concentration per day for 10 blocks), mineral oil serving as S−. The concentrations of odorant used in these tasks were 1%, 0.1%, 0.01%, 0.001% and 0.0001% (vol/vol). Two odorants were used: (+) carvone for SSTR4 cohort and isoamylacetate for SOM and SSTR2 cohorts.

Next, animals were trained to discriminate between mixtures with increasing complexity to evaluate discrimination acuity. Mice were first trained to distinguish between two similar odorants [(+) and (−) carvone enantiomers], (+) carvone being used as S+. Then, animals had to discriminate between progressively closer binary mixtures of enantiomers where (+) carvone proportion in S+ was progressively decreased from 80% to 52.5%. Thus, the concentration of (+) carvone/(−) carvone enantiomers in the mixture was sequentially equalized in separate sessions (one session per day, 10 blocks per session) to 80/20, 60/40 and 52.5/47.5 for S+ vs. 20/80, 40/60 and 47.5/52.5 for S−, respectively.

Twenty-one days after the initiation task, in addition to Discrimination 3, mice were tested for olfactory memory of the initiation discrimination task. The 20 trials of each block were composed of 16 trials for the enantiomer discrimination and four trials for the olfactory memory in which no reward was given (two hexanal and two heptanal for SSTR4 cohort, two anisole and two cineole for SOM and SSTR2 cohorts). Memory performance was calculated from the averaged performances in these four trials.

Data were expressed as mean percentage of correct response for each training block. Five-block data were averaged to analyze learning performances. The performances of the last three blocks of a training session were averaged and this mean value was used as a discrimination score for each group.

### Statistical Analysis

All results are expressed as mean ± standard error of the mean (SEM). The degree of statistical significance was calculated using STATVIEW software (SAS Institute, Cary, NC, USA). For SSTR2 distribution, two-way repeated-measures analysis of variance (r-m ANOVA) with cellular layer as an in-between factor and anteroposterior levels as a within-subjects factor with Bonferroni correction was used.

Statistical analyses for behavioral data can be found in Supplementary data. Raw data of the habituation-dishabituation protocol were analyzed, respectively between H1 and H4 trials, H4 and C_+1_ trials and H5 and C_+3_ trials using two-way r-m ANOVA with the group as in-between subjects factor and trials as within-subjects factor. For multiple comparisons, a Bonferroni *post hoc* test was performed.

For olfactory operant behavioral protocols, BTC, memory and final performance data (mean of the last three blocks) were analyzed using one-way ANOVA with the group as a between-subjects factor. Learning or session performances were analyzed using two-way r-m ANOVA with the group as between-subjects factor and trials, blocks as within-subjects factors. The effect of concentration (or complexity) on performances was analyzed using three-way r-m ANOVA using S+ concentration (or mixture) as an additional within-subjects factor. For multiple comparisons, a Bonferroni *post hoc* test was performed.

## Results

### Cellular Distribution of SOM Systems in the Main Olfactory Bulb

Since molecular and binding studies had shown the abundance of SSTR1-SSTR4 subtypes in mouse OB, we validated and used a combination of immunological tools (Table 1) and transgenic mice models to study the cellular localizations of SOM peptide, SSTR2, SSTR3 and SSTR4 receptors (Figure 1). SSTR1 distribution was not attempted because poor specificity was found for the available SSTR1 antibodies using SSTR1 knockout mice (Kreienkamp et al., 1999), see also https://www.abcam.com/Somatostatin-Receptor-1-antibody-ab100881/reviews/39250). As previously reported in Lepousez et al. (2010a), SOM is mainly expressed in interneurons of the inner part of the EPL, as well as running fibers and sparse deep short axon cells in the GCL (Figures 1A–C), Interestingly, SSTR2, SSTR3 or Beta-galactosidase (β-Gal)-mediated SSTR4 patterns delineated distinct bulbar layers, from the GCL to the peripheral GL (see Figures 1A–C and Figures 5B,E,H).

Monoclonal anti-SSTR2 antibody mainly labeled small neurons located in the mitral cell layer (MCL) with typical dendrite-like projections crossing the EPL and projecting into the GL (magenta, Figures 1A, 2). Most cells showed small round cell bodies (mean diameter 9.25 ± 0.19 μm, *n* = 232 cells, *N* = 2) but colocalization was not found with SOM (Figures 1A, 5C) or with interneuron markers parvalbumin (Figure 2A) or calretinin (Figures 2B, 3A–C). SSTR2-positive cells were intermingled with the mitral cells, recognizable by their large pear-shaped somata (mean diameter 16.97 ± 1.30 μm) and thick primary dendrites (Figures 2D,G). SSTR2-positive cells never co-expressed the mitral cell markers reelin, Tbx21 or Kv3.1-EYFP markers (Figures 2D,G, enlarged in Figures 2E, 3D–F). Reelin also labels tufted cells in the outer EPL, but no SSTR2-labeled cells were found at this level (Figures 2D,G). We cannot exclude a minimal expression of reelin in some MCL SSTR2 cells since a faint signal was occasionally detected (Figures 2E, 3E,F). At any rate, SSTR2 labeling did not colocalize with GAD67 fibers and cell bodies (Figures 2I, 3G–I), suggesting that OB SSTR2 neurons are not GABAergic. SSTR2 projections delineated glomeruli, some of them being strongly labeled, and overlapped with OMP labeling without colocalization (Figure 2C, enlarged in Figure 2F). SSTR2 density significantly decreased along the anteroposterior axis (sampled every 250 μm caudally until Bregma 4.25; percent SSTR2 stained area: GL *F*_(5,17)_ = 3.070, *P* = 0.037, EPL *F*_(5,17)_ = 3.702, *P* = 0.019, GCL *F*_(5,16)_ = 0.204, *P* = 0.96) showing enrichment in SSTR2 glomeruli in the rostral part of the bulb while SOM density did not significantly change (percent SOM stained area: GL *F*_(5,16)_ = 0.303, EPL *F*_(5,16)_ = 0.989, GCL *F*_(5,16)_ = 0.564, *P*s > 0.4; Figure 4). In the inner layers IPL (internal plexiform layer, just below the MCL) and GCL, a dense and fine network of SSTR2 fibers was observed (Figures 1A, 2A–D). Occasionally some SSTR2-positive cells in the IPL had lateral dendritic projections, and strongly labeled superficial short-axon-like cells were observed in the GCL. A similar pattern (Figure 5A) was observed after β-Gal labeling in homozygous SSTR2 KO-lacZ KI mice (Figure 5I), and SSTR2 labeling totally disappeared in SSTR2 KO and SSTR2 KO-LacZ KI homozygous animals (Figures 5D,G).

SSTR3 antibody labeled typical primary cilia patterns in the OB, as reported in many brain regions (O’Connor et al., 2013), SSTR3 signals were sparse in the GL and highly concentrated in the IPL and GCL mirroring the dense distribution of cells (mostly granule cells) in these layers (Figures 1B, 6A). In the GCL, all SSTR3-positive cilia were also labeled with the ciliary marker Arl13b antibody (Figure 6A, zoom in Figure 6B). As a comparison, primary cilia positive for the ciliary marker adenylyl cyclase III was more abundant in the GL and EPL layers (Figure 6C).

Since no commercially available SSTR4 antibody showed reliable specificity, β-Gal expression was used to localize SSTR4-expressing cells in heterozygous or homozygous SSTR4 KO-LacZ KI mice (Helyes et al., 2009). β-Gal nuclear expression was predominantly found in cells surrounding the glomeruli in the GL and sparsely disseminated in the GCL (Figures 1C, 6D). Among the main known periglomerular cell populations (Nagayama et al., 2014), β-Gal antibody specifically labeled the nuclei of approximately a third of the calretinin-positive population (Figure 3E, 32.5% ± 1.7, *n* = *N* = 48 sections for four animals) and did not colocalize with TH, calbindin nor parvalbumin (not shown). Among calretinin-positive cells, β-Gal nuclear staining was associated with nNOS-expressing neurons (Figures 6F, 3J–L). Both double staining of β-Gal with CR or nNOS antibody showed predominant intraglomerular projections (Figures 6E,F).

### Main Afferents to Bulbar Somatostatinergic Populations

Since retrograde afferents are known to modulate bulbar synaptic activity, we decided to map the neural afferents targeting bulbar somatostatinergic cells. A conditional pseudorabies virus expressing GFP (PRVBa2001, DeFalco et al., 2001) was injected in the OB of SOM-Ires-Cre heterozygous mice, together with fluorescent beads to visualize the injection site. Mice were sacrificed 3–5 days after infection and the pattern of GFP expression was examined in serial sections of the whole brain at 3 days post-injection (3 dpi; Figure 7). GFP-expressing cells were mainly found in the olfactory cortical area, i.e., the AON, piriform and entorhinal cortex, with rare cells occurring in the dorsal tenia tecta (DTT), and the posteromedial cortical amygdala (PMCo). The number of labeled neurons increased with time in these regions (see Figures 7, 8), which send monosynaptic inputs to the OB (Shipley and Ennis, 1996; Mohedano-Moriano et al., 2012; De La Rosa-Prieto et al., 2015; Diodato et al., 2016). Stronger retrograde infection by the virus appeared after 5 dpi in extra-olfactory regions, the ventral CA1 of the hippocampus, the claustrum, the paraventricular nucleus of the hypothalamus (not shown), the agranular insular cortex, the basolateral amygdala (BLA) and the locus coeruleus (LC; Figure 8). Except for the LC (Shipley and Ennis, 1996; Schwarz et al., 2015), these regions have not been identified as direct projection areas to the OB (Shipley and Ennis, 1996; Diodato et al., 2016) and the late detection of GFP suggests that they are second- or higher-order projection neurons to the OB (Figure 8), consistent with results using different tracing methods (Shipley and Ennis, 1996; Mohedano-Moriano et al., 2012; De La Rosa-Prieto et al., 2015; Diodato et al., 2016). These data suggest that higher cortical centers modulate SOM signaling in the OB.

### Impact of SOM Transduction Impairment on Olfactory Performances

The olfactory performances of WT and KO mutant littermates were compared using a sequence of behavioral tests (Table 2). The respective impact of SOM, SSTR2 and SSTR4 removal was studied separately, using dedicated transgenic mice cohorts.

In the habituation/dishabituation protocol, mice were exposed four times to a first odorant stimulus (habituation odor H), then sequentially to test odors of variable similarity with respectively one (C + 1) or three (C + 3) additional carbon chains.

In the SOM cohort (Figure 9A), odor exploration significantly decreased over time between H1 and H4 trials (*F*_(3,36)_ = 16.95, *P* < 0.0001, r-m ANOVA) suggesting global odor habituation since no significant group effect or trial × group interaction were found (group: *F*_(1,12)_ = 0.67, *P* > 0.05; trial × group interaction *F*_(3,36)_ = 0.17, *P* > 0.05). Exploration time increased between H_4_ and C_+1_ trials (*F*_(1,12)_ = 10.29, *P* < 0.01) and between H5 and C_+3_ trials (*F*_(1,12)_ = 68.25, *P* < 0.0001), indicating that mice discriminate between the habituated odor and the test odors. No significant group effect or trial × group interaction was found regardless of test odor (H4 vs. C_+1_: group (*F*_(1,12)_ = 0.80, trial × group interaction *F*_(1,12)_ = 0.12; H5 vs. C_+3_: group: *F*_(1,12)_ = 3.75, trial × group interaction *F*_(1,12)_ = 0.20, all *P*s > 0.05).

In the SSTR2 cohort (Figure 9B) exploration globally decreased between H1 and H4 trial in all mice (Trial: *F*_(3,39)_ = 6.19, *P* < 0.01) similarly in WT and SSTR2 KO groups (Group: *F*_(1,12)_ = 0.67, *P* > 0.05, no group × trial interaction) suggesting odor habituation. Mice investigated more the habituated odor than the test odors (Trial H_4_ vs. C_+1_: *F*_(1,13)_ = 6.68, *P* < 0.05, Trial H_5_ vs. C_+3_ *F*_(1,13)_ = 4.69, *P* < 0.05) but no significant group effect was found (Group: H_4_ vs. C_+1_ *F*_(1,13)_ = 0.37, *P* = 0.55, H_5_ vs. C_+3_ *F*_(1,13)_ = 3.04 *P* = 0.10, *P*s > 0.05).

In the SSTR4 cohort (Figure 9C) odor exploration strongly decreased between H1 and H4 for WT and SSTR2 KO groups (trial: *F*_(3,42)_ = 38.66, *P* < 0.0001), indicating habituation in both groups (Group *F*_(1,14)_ = 2.480, *P* = 0.14). Mice investigated more C_+1_ than H4 (Trial: H_4_ vs. C_+1_ *F*_(1,14)_ = 84.38, *P* < 0.0001), SSTR4 KO mice exploring significantly longer than WT (group: *F*_(1,14)_ = 5.26, *P* < 0.05). Between H5 and C_+3_, odor exploration time increased similarly in both groups (Trial: *F*_(1,14)_ = 40.17, *P* < 0.0001, group: *F*_(1,14)_ = 0.06, *P* > 0.05).

Next, WT and KO littermates were submitted to olfactory operant conditioning to compare their fine olfactory performances (see Table 2 for details). During this task, the mice have to lick when presented with a rewarding odor S+ and not to lick when the non-rewarding odorant S− is presented (correct responses). For the sake of clarity, results independently obtained for each cohort are reported together, task per task.

Mice were first taught to learn the rule and to discriminate dissimilar odor pairs in an initiation task (Table 2). As illustrated in Figure 10A, performances progressively increased with training showing that mice learned to correctly discriminate the anisole-cineole odor pair (5-block: SOM cohort, *F*_(6,66)_ = 24.08, *P* < 0.0001 *n* = 6–7; *SSTR2* cohort, *F*_(5,65)_ = 60.06, *P* < 0.0001, *n* = 7–8). No group effect was observed (group: SOM cohort *F*_(1,11)_ = 1.35, SSTR2 cohort *F*_(1,13)_ = 0.72, *P*s > 0.05). SSTR4 mice required 50 blocks to improve their performances with hexanal/heptanal odor pair (5-blocks:* F*_(9,108)_ = 17.29, *p* < 0.0001, *n* = 7), similarly in WT and SSTR4 KO (group: *F*_(1,12)_ = 2.16, *P* > 0.05). The number of blocks necessary to reach the 85% learning criterion (BTC) was not significantly different between WT and KO mice for each cohort (Figure 10B), suggesting that learning the rule was not affected in any mutant mice (group: SOM cohort *F*_(1,12)_ = 2.19, SSTR2 cohort *F*_(1,13)_ = 0.52, SSTR4 cohort *F*_(1,12)_ = 1.84, all *P*s > 0.05, Bonferroni-corrected ANOVA, *n* = 7–8). In each cohort, performances after learning (mean of the last three blocks) were similar in WT and KO mice (group: SOM cohort: WT = 84.29 ± 2.83, SOM KO = 85.23 ± 3.09, *F*_(1,12)_ = 0.02; SSTR2 cohort: WT = 86.45 ± 2.63, SSTR2 KO = 89.05 ± 2.00, *F*_(1,13)_ = 0.27; SSTR4 cohort: WT = 75.00 ± 3.93, KO = 84.52 ± 3.68, *F*_(1,12)_ = 1.04, all *P*s > 0.05, Bonferroni-corrected ANOVA, *n* = 7–8). Twenty-one days after completion of the task, mice were tested for memory of this initial discrimination (Figure 10C). In each cohort, no significant difference was found between WT and KO mice (group: SOM cohort *F*_(1,12)_ = 0.06; SSTR2 cohort *F*_(1,13)_ = 1.14; SSTR4 cohort *F*_(1,12)_ = 0.19; all *P*s > 0.05, Bonferroni-corrected ANOVA, *n* = 7–8), suggesting that SOM, SSTR2 or SSTR4 deletion did not affect olfactory memory after a 3-week delay.

Next, olfactory detection was evaluated by measuring how mice discriminate serial dilutions of an odorant. Mice were first trained to discriminate a novel S+ odor from its solvent (mineral oil; Table 2) until they reached the 85% learning criterion. WT and KO mice reached similar performances at the end of the task in each cohort (mean of the last three blocks: group: SOM cohort WT = 93.6 ± 2.2%, SOM KO = 81.9 ± 5.4%, *F*_(1,12)_ = 0.26, *n* = 7; SSTR2 cohort WT = 90.8 ± 4.9%, SSTR2 KO = 85.2 ± 6.1%, *F*_(1,13)_ = 2.56, *n* = 8–7; SSTR4 cohort WT = 92.4 ± 1.5%, SSTR4 KO = 90.9 ± 2.9%, *F*_(1,12)_ = 0.19, *n* = 7; all *P*s > 0.05, Bonferroni-corrected ANOVA). Then mice were exposed to decreasing series of S+.

In the SOM cohort (Figure 10D_1_), ANOVA performed on all training sessions showed a significant effect of concentration on performance (*F*_(4,48)_ = 9.13 *P* < 0.0001), suggesting that performances decreased over concentrations in both WT and SOM KO mice with no group effect (group: *F*_(1,12)_ = 1.54, *P* > 0.5) but a significant concentration × group interaction (*F*_(4.48)_ = 4.023, *P* < 0.01). ANOVA performed on each training session did not show any significance between groups (*P*s > 0.05). Analysis of the last three blocks (Figure 10E_1_) showed that averaged performance significantly decreased with S+ concentration (concentration: *F*_(4,48)_ = 52.64, *P* < 0.0001), WT performing always better than SOM KO mice (group: *F*_(1,12)_ = 4.90, *P* = 0.05, no significant interaction).

In the SSTR2 cohort (Figure 10D_2_), ANOVA performed on all training sessions showed a significant effect of concentration on performance (*F*_(4,52)_ = 22.39, *P* < 0.0001) and no significant group effect (*F*_(1,13)_ = 4.58, *P* = 0.052) or significant interaction. Analysis of the mean of the last three blocks (Figure 10E_2_) showed significant concentration and group effects (concentration: *F*_(4,52)_ = 11.33, *P* < 0.0001, group: *F*_(1,13)_ = 4.86, *P* < 0.05) and a significant concentration × group interaction (*F*_(4,52)_ = 2.90, *P* < 0.05) indicating that performances of WT and SSTR2 KO mice evolved differentially over S+ dilutions. *Post hoc* analysis showed that SSTR2 KO mice scores were significantly lower than WT at 0.1%, 0.01% and 0.001% (group: *F*_(1,13)_ = 9.62, *P* < 0.01, *F*_(1,13)_ = 5.95, *P* < 0.05, *F*_(1,13)_ = 11.41, *P* < 0.01, respectively) but not at 1% (*F*_(1,13)_ = 0.53, *P* > 0.05) or 0.0001% (*F*_(1,13)_ = 0.06, *P* > 0.05, Figure 10E_2_).

In the SSTR4 cohort, ANOVA performed on all training sessions showed a significant effect of concentration (*F*_(3,36)_ = 58.52, *P* < 0.0001) without any group effect (*F*_(1,12)_ = 0.0003, *P* > 0.05) or significant interaction, indicating that performances globally decreased for both groups. Mean of the last three blocks (Figure 10E_3_) decreased with concentration (*F*_(3,36)_ = 60.48, *P* < 0.0001) similarly in WT and SSTR4 KO mice (group: *F*_(1,12)_ = 0.01, *P* > 0.05, group × concentration interaction *F*_(3,36)_ = 0.40, *P* > 0.05).

In summary, while SOM KO showed lower performances than WT, only SSTR2 KO mice showed significantly impaired detection responses in our experimental conditions.

Finally, olfactory discrimination acuity was evaluated by increasing the complexity of the task using binary mixtures of two enantiomers, (+) carvone and (−) carvone (Table 2). Mice first learned to discriminate between pure carvone enantiomers (1% vol/vol). In each cohort, WT and KO mice reached similar performances at completion of the task (averaged last three blocks: group: SOM cohort WT = 86.3 ± 6.2%, SOM KO 84.8 ± 7.6%, *F*_(1,12)_ = 0.05, *n* = 7; SSTR2 cohort WT 94.4 ± 1.5%, SSTR2 KO 86.7 ± 2.7%, *F*_(1,13)_ = 2.97, *n* = 8–7; SSTR4 cohort WT 95.7 ± 1.3%, SSTR4 KO 92.9 ± 1.8%, *F*_(1,12)_ = 0.93, *n* = 7, all *P*s > 0.05). All groups reached the criterion, showing that they discriminate the two odorants. Mice were then expected to progressively discriminate between 100/0, 80/20, 60/40, 55/45 and 52.5/47.5 binary mixtures of 1% (+) carvone and 1% (−) carvone, such as the difficulty of the discrimination task increased over sessions.

In the SOM cohort (Figure 10F_1_). ANOVA on all training sessions showed that WT and KO discrimination performances globally decreased with task complexity (mixture: *F*_(3,36)_ = 30.93, *P* < 0.0001), similarly in both groups (group: *F*_(1,12)_ = 0.01, *P* > 0.05, no significant interaction). Analysis of the averaged last three blocks (Figure 10G_1_) confirmed that discrimination performances significantly decreased with task difficulty (mixture: *F*_(3,36)_ = 29.02, *P* < 0.0001) similarly in both groups (group: *F*_(1,12)_ = 0.001, *P* > 0.05, no significant interaction).

In the SSTR2 cohort (Figure 10F_2_), ANOVA on all training sessions showed that global performances decreased with task complexity (mixture: *F*_(3,39)_ = 66.52, *P* < 0.0001) with a significant group effect (*F*_(1,13)_ = 5.20, *P* < 0.05, no significant interaction). Analysis of each session showed that SSTR2 KO performed significantly lower than WT at 80/20 and 60/40 mixtures (group: *F*_(1,13)_ = 5.51, *P* < 0.05 and *F*_(1,13)_ = 5.26, *P* < 0.05). Mean of the last three blocks (Figure 10G_2_) analysis showed that final performances decreased with task complexity (mixtures: *F*_(3,39)_ = 32.27, *P* < 0.0001) in both groups (group: *F*_(1,13)_ = 9.68, *P* < 0.01, no significant interaction). Analysis of each session showed that SSTR2 KO mice performed significantly lower than WT at 80/20 (*F*_(1,13)_ = 7.12, *P* < 0.05) and 60/40 (*F*_(1,13)_ = 5.25, *P* < 0.05; Figure 10G_2_) while there was no group difference at 100/0 and 55/45 (*P*s > 0.05).

In the SSTR4 cohort, discrimination performances also globally decreased with task complexity (mixture: *F*_(3,36)_ = 40.28, *P* < 0.0001) without significant difference between WT and KO mice (group: *F*_(1,12)_ = 0.32, *P* > 0.05, no significant interaction). Averaged last three blocks performance (Figure 10G_3_) showed that WT and SSTR4 KO discrimination scores similarly decreased in both groups (mixture: *F*_(3,36)_ = 22.66, *P* < 0.0001, group: *F*_(1.12)_ = 0.041, *P* > 0.05, no significant interaction).

In summary, SSTR2 KO mice showed impaired discrimination performances as compared to WT when the discrimination task was getting difficult in our experimental conditions.

## Discussion

In this work we combined histological, viral tracing and transgenic approaches to characterize the cellular targets of SOM in the murine OB. We demonstrate that SOM targets all levels of mitral dendritic processing in the OB with SSTR2 being expressed on the soma and dendrites of previously uncharacterized mitral-like cells, SSTR4 being associated with inhibitory periglomerular cells in the GL and SSTR3 restricted to neuronal cilia concentrated in the GCL. Genetic deletion of SSTR4, SSTR2 or SOM differentially affected olfactory performances. SSTR4 deletion did not impact the olfactory phenotype. Olfactory detection was differentially impaired in SOM KO and SSTR2 KO mice while only SSTR2 KO mice showed impaired fine discrimination. These data bring novel neuroanatomical and functional arguments in favor of the fine modulation of olfactory functions by SOM and call for future studies dissecting the respective origin and contribution of each SSTR subtype in the cellular and physiological responses to the peptide during olfactory processing.

One important finding was the identification of an atypical mitral cell-like neuronal population identified by SSTR2 expression. Using a specific monoclonal SSTR2 antibody, we show that SSTR2 labels a subpopulation of ovoid cells of the MCL layer projecting a single thick dendrite toward the GL. These cells are intermingled with the large typical mitral cells recognizable with their pear-shaped soma (>20 μm, Nagayama et al., 2014) and thick primary dendrites labeled with Kv3.1 or Tbx21 antibodies. Further, SSTR2 is not detected in tufted cells. Some SSTR2-expressing neurons faintly express reelin in their soma, a marker of mitral and tufted cells in the OB while no colocalization was found with GABAergic or interneuron markers. SSTR2-positive cells strongly innervate glomeruli in the GL and interact with OMP-positive compartments (Nagayama et al., 2014). Thin labeled axon-like neurites are visible in the GCL, but the lateral olfactory tract is not labeled, suggesting that SSTR2 is mainly localized in somatic and dendritic compartments of principal cells, as previously reported in most forebrain structures (Csaba and Dournaud, 2001; Viollet et al., 2008; Liguz-Lecznar et al., 2016). Our study thus biochemically identifies the SSTR2-positive cells previously observed after agonist internalization using a polyclonal anti-SSTR2 antibody (Lepousez et al., 2010a). In this latter study, SSTR2 labeling colocalized with dextran staining after retrograde injection of the retrograde tracer into the GL, showing that SSTR2-positive cells did project to the GL, consistent with the labeling of large dendrites in the EPL during ligand internalization (Lepousez et al., 2010b). Altogether, our data strongly suggest that SSTR2 labeling reveals a subpopulation of non-GABAergic mitral-like cells, not yet reported (Nagayama et al., 2014). Additionally, we found some SSTR2-positive cells in the IPL with lateral dendritic projections, and strongly labeled superficial short axon-like cells in the GCL. Such a SSTR2 immunohistochemical distribution in the OB is found in WT mice as well as in SSTR2KO-lacZ-KI mutant labeled with β-Gal antibody (Allen et al., 2003) and it disappears in homozygous SSTR2 KO or SSTR2 KO-LacZ KI mutant mice as expected.

Interestingly, not only SSTR2 but also SSTR1, SSTR3 and SSTR4 subtypes are highly expressed in the OB and display distinct binding patterns, each being associated to a given OB layer (Videau et al., 2003; Martel et al., 2015). We confirm here that SSTR3 and SSTR4 are associated to GCL and GL layers, respectively. SSTR3 is associated to the primary cilium, considered as a chemical sensor due to its concentration of many GPCRs and associated transduction pathways. Its loss leads to major cognitive and physiological defects (Louvi and Grove, 2011). In the OB, SSTR3 colocalizes with the ciliary marker Arl13b whose staining reflects nuclear density in all layers. SSTR3-labeled cilia are dense in the GCL, suggesting they may be associated with packed inhibitory granule cells which constitute 90% of OB neurons. We cannot exclude that they also label glial cells, but SSTR3 staining was associated with NeuN-positive cells in most brain telencephalic regions when studied in detail (Sipos et al., 2018). Recent studies in striatal interneurons has indicated that the major role of ciliary SSTR3 is in the maintenance of cellular connectivity and synaptic activity (Guo et al., 2017), showing that ligand-receptor interaction has crucial consequences for cellular function (Green et al., 2016; Nager et al., 2017). Furthermore, pharmacological and genetic evidence has demonstrated that knocking-out *sstr3* gene in the hippocampus affects synaptic plasticity and recognition memory (Einstein et al., 2010). Since granule cells are major actors in the synaptic modulation of the olfactory signal in the OB through the reciprocal dendritic regulation of mitral cells, SSTR3 activation may directly influence their synaptic activity as well as their responses to local inhibition (Nusser et al., 2001) or afferent modulatory inputs (Nunez-Parra et al., 2013) which are proposed to control oscillatory states (David et al., 2015).

An intriguing finding was also that SSTR4 labeling is mostly associated to the GL in a nNOS-positive calretinin-positive subpopulation of GABAergic periglomerular cells (Kosaka and Kosaka, 2005; Nagayama et al., 2014). Due to technical constraints including a lack of specific SSTR4 antibody and nuclear expression of beta-galactosidase in the SSTR4 KO-LacZ KI (Helyes et al., 2009), we relied on nNOS immunohistochemical properties to conclude that cells bearing SSTR4 mainly project to the intraglomerular domain (Crespo et al., 2003; Nagayama et al., 2014). In such a way, SSTR4 SOM receptors are positioned to modulate early synaptic stages of olfactory processing, either directly or through the release of nNOS, a potent neuromodulator in other brain structures (Vasilaki et al., 2002; Mastrodimou et al., 2006; Pavesi et al., 2013; Liguz-Lecznar et al., 2016). Furthermore, a functional interaction occurs between two compartment-based SSTR2 and SSTR4 receptor subtypes in the mouse hippocampus (Moneta et al., 2002; Gastambide et al., 2010). In the OB, SSTR4-expressing cells surround glomeruli labeled by SSTR2-positive terminals suggesting a similar mechanism. The physiological conditions requiring SSTR4 activation remain obscure. One hypothesis would be that SSTR4 plays a role in the olfactory control of emotional behavior, since several studies have consistently reported anatomical, physiological and behavioral evidence that deleting *sstr4* gene induces stress responses in mice (Scheich et al., 2016; Prévôt et al., 2017). The local or extra-bulbar origin of the ligand targeting these receptors remains an open question since, no major SOM immunostaining was detected in the GL or OSNs, as previously reported (Lepousez et al., 2010a; De La Rosa-Prieto et al., 2016). Only rare somata and parts of SOM neurites were occasionally labeled in the GL, in accordance to *in situ hybridization* data in the GL from Allen Brain Atlas^1^. However, it remains possible that under special conditions a surge in SOM release is triggered to activate SSTR4 receptors. Another possibility involves cortistatin, a peptide homologous to SOM which binds to SSTR receptors with equivalent affinities (Martel et al., 2012). Cortistatin has a more restricted brain distribution than SOM (de Lecea, 2008) and has been described in rat and mouse OB where it is expressed at low levels, like SOM (Martel et al., 2015). *In situ* hybridization data^2^ and distribution of Cre or GFP tracers in cortistatin transgenic model mice^3^ confirm that CST is mainly expressed in MCL and outer GCL cells which project to the outer border of the EPL, providing an appropriate alternate source of SOM-like ligand in the OB.

Finally, SSTR1 is highly expressed in the OB (Martel et al., 2015) suggesting that SSTR1 may add another modulatory level, including an autoreceptor function as previously described in other forebrain structures (Thermos et al., 2006; De Bundel et al., 2010; Martel et al., 2012). No home-made or commercial SSTR1 antibody tested could be properly validated using an SSTR1 KO strain (Kreienkamp et al., 1999, data not shown) to be used to map the cells expressing this subtype in the OB.

According to that scheme, SSTR subtypes are differentially expressed in neurons involved in distinct inhibitory circuits regulating the dendritic excitability of the mitral cells in the OB. SOM cells may thus constitute a core of a sub-circuit in the OB, where the peptide exerts neuromodulatory influences through the dendritic regulation of afferent and recurrent excitation in principal cells and/or inhibition in GABAergic interneurons, as previously shown in piriform and neocortical circuits (Sturgill and Isaacson, 2015; Large et al., 2016). SOM neuromodulation may partly result from centrifugal influence occurring through GCL (and occasionally GL) SOM fibers, originating from AON as shown here or from the medial raphe nucleus (Araneda et al., 1999), and the piriform cortex (Diodato et al., 2016). Comparison of rabies-based Cre-dependent retrograde viral infection in SOM-Ires-Cre mice with published evidence reporting monosynaptic inputs to the OB (Shipley and Ennis, 1996; Diodato et al., 2016) shows that OB SOM populations themselves receive direct centrifugal projections from a restricted number of central structures. Discrete groups of GFP-labeled neurons are found after 3 dpi in the AON, dorsal tenia tecta (DTT), piriform and entorhinal cortex, medial amygdala and ventral CA1 of the hippocampus, all structures involved in olfactory and emotional processing and previously reported to send back projections to the OB (Hintiryan et al., 2012; Miyamichi et al., 2013; Diodato et al., 2016). Most centrifugal projections are glutamatergic but basal forebrain nuclei send cholinergic fibers as well as GABAergic fibers to the OB (Nunez-Parra et al., 2013; Case et al., 2017; Sanz Diez et al., 2017) which may mediate disinhibition of the principal cells (Gracia-Llanes et al., 2010). Progressively, GFP infected neurons appear in the hypothalamic paraventricular nucleus, claustrum and BLA, suggesting that the virus retrogradely infects second- or third-order neurons. Indeed these regions, except the LC, are not known as direct projection areas to the OB (Shipley and Ennis, 1996; Mohedano-Moriano et al., 2012; De La Rosa-Prieto et al., 2015; Diodato et al., 2016). In conclusion, SOM populations in the mouse OB are tightly contacted by top-down afferents, most of them coming from regions involved in olfactory and emotional processes.

Such occurrence of centrifugal afferents on somatostatinergic modulation in the OB may explain why the genetic deletion of SSTR2 or SSTR4 receptor or SOM peptide, which are expressed at key levels of olfactory processing in the OB, leads to contrasted phenotypes after olfactory evaluation. A consistent set of data suggests that the OB is the primary site for odor detection and recognition (Uchida and Mainen, 2003; Wesson et al., 2008), filtering the information coming from olfactory sensory neurons. In mice, a single respiratory sniff allows the discrimination of a novel odor, based on immediate glomerular activation (Chong and Rinberg, 2018) and local changes in principal cells activity (Sirotin et al., 2015). A coordinated firing of many neurons across the OB would mediate local gamma oscillations, whose power is correlated to fine discrimination performances (Kay, 2014) and integrative properties of neuronal ensembles with intracortical associational synapses in the piriform cortex are involved in the decoding of odor features. The piriform cortex is involved in the discrimination of simple tasks, based on spike timing and synchrony of local field potentials oscillations in the gamma band but also in the beta band which emerge with learning and experience in every part of the olfactory system (Kay, 2014; Wilson et al., 2014). More difficult discrimination between close or complex odors would mainly engage top-down inputs from the entorhinal cortex, responsible for pattern separation processes (Chapuis et al., 2013; Wilson et al., 2014). Finally, afferents from the orbitofrontal cortex seem preferentially involved in the reward–value of an odor and long-term memory encoding in the piriform cortex (Wilson et al., 2014).

Using a spontaneous olfactory discrimination task, we found that WT and KO mice behaved similarly in SOM, SSTR2 and SSTR4 cohorts concerning the habituation to an odor or the discrimination of a novel odorant (H_5_ vs. C_+3_), suggesting that deleting these genes has no major impact on short-term olfactory memory formation or odor discrimination abilities in our conditions. Complementary experiments varying odorants at lower concentrations may reveal specific roles, if any, of SOM, SSTR2 and SSTR4 on short-term memory.

Concerning SSTR2, we had previously shown that its pharmacological blockade or activation in the OB respectively impaired or improved fine olfactory discrimination. Discrimination performances were correlated with power changes in gamma oscillations recorded in the OB of awake mice (Lepousez et al., 2010b). We show here that SSTR2 gene deletion affects olfactory performances in an operant task. since the mice fail to reach the discrimination criterion earlier than WT when the difficulty of the task increases. In line with our previous pharmacological data, this supports the hypothesis involving SSTR2 receptors and endogenous SOM in the modulation of olfactory discrimination and basal gamma oscillations in the OB. Since gamma oscillations rely on dendrodendritic synaptic interactions between mitral and granule cells, SSTR2 receptors may mediate a potent endogenous somatostatinergic tone on the mitral-like cells of the OB described herein. Reciprocal synapses between SOM interneurons and mitral dendrites have been previously reported (Lepousez et al., 2010a), but the ultrastructural localization of SSTR2 receptors at this level has not been described yet. Furthermore, since the SSTR2 KO mouse line is a constitutive transgenic line, we cannot exclude that the removal of SSTR2, present at all levels of the olfactory pathway, especially in both piriform and entorhinal cortex (Allen et al., 2003; Martel et al., 2015) also impacts the discrimination of very similar odors. Interestingly we show here that SSTR2 deletion also impairs olfactory detection abilities in an operant task. Alteration of both fine discrimination and detection was previously reported in mice lacking mitral but not tufted cells (Díaz et al., 2012), in agreement with the exclusive detection of SSTR2 in mitral-like cells in the OB. In comparison, removing SOM has few effects on olfactory detection (and no effect on discrimination) in our experimental conditions. It is somehow counterintuitive that removing the peptide has less effect than removing one single receptor out of four in the OB. This may be due to a global redistribution of the receptors, since a massive up-regulation of SOM binding sites is observed in SOM KO mice (Videau et al., 2003) and *in vivo* and *in vitro* data showed that intracellular localization and trafficking of all SSTR except SSTR4 is strongly dependent on SOM release in physiological or pathophysiological conditions (Csaba and Dournaud, 2001; Le Verche et al., 2009; Csaba et al., 2012). Another explanation would rely on the extent of redundancy between SOM and cortistatin peptides in the olfactory pathway since both peptides exert distinct cellular and functional effects in the cortex (de Lecea, 2008).

Finally, SSTR4 KO and WT animals displayed similar olfactory behavioral responses in our experimental conditions. This was unexpected considering the abundance of SSTR4 binding sites (Martel et al., 2015) and SSTR4-expressing periglomerular cells at the first synaptic crossroad in the OB where odor detection and contrast enhancement takes place (Wilson et al., 2014; Chong and Rinberg, 2018). No major change in habituation, learning, detection or discrimination abilities was observed in the SSTR4 KO mice when compared to WT littermates. Since this receptor induces hyperpolarizing synaptic effects (Qiu et al., 2008), periglomerular SSTR4 may be required in given physiological conditions inducing a strong local release of somatostatinergic ligands (SOM or cortistatin). The question of the physiological conditions requiring SSTR4 activity remains to be addressed.

In conclusion, this anatomical and behavioral study opens novel perspectives concerning the modulatory roles of SOM in mouse OB. Previous pharmacological results (Lepousez et al., 2010b) and the transgenic data included here show that bulbar SOM, either endogenous or released from centrifugal afferents, exerts a tonic control on the activity of SSTR2-positive mitral cells in the OB. It also suggests more complex regulations involving different SSTR subtypes and additional olfactory regions. Physiological studies with opto- and chemogenetic models are now clearly required to dissect the contribution of each peptide and SSTR subtype in the synaptic modulatory effects of SOM in olfactory processing.

## Author Contributions

CV and JE designed research. AS and SN performed immunohistochemistry. JG, FD and NS viral tracing. SN, OF, YC and CV behavioral experiments. CV prepared the manuscript.

## Conflict of Interest Statement

The authors declare that the research was conducted in the absence of any commercial or financial relationships that could be construed as a potential conflict of interest.

## Footnotes

^1^http://mouse.brain-map.org/gene/show/20366

^2^http://mouse.brain-map.org/experiment/show?id=72472766

^3^http://www.gensat.org/imagenavigator.jsp?imageID=66192

## Abbreviations

AON: anterior olfactory nucleus
BTC: blocks to criterion
Ent: entorhinal cortex
EPL: external plexiform layer
GL: glomerular layer
GCL: granule cell layer
OB: olfactory bulb
Pir: piriform cortex
Post: posterior
SOM: somatostatin
SSTR: somatostatin receptor.
