# Supplementary material for: Somatostatin Serves a Modulatory Role in the Mouse Olfactory Bulb: Neuroanatomical and Behavioral Evidence
Source: Front Behav Neurosci. 2019 Apr 9;13:61. doi: 10.3389/fnbeh.2019.00061 (PMC6465642; doi:10.3389/fnbeh.2019.00061)
Supplement: Supplementary file 1 [file Data_Sheet_1.pdf]

**Statistical effects of genotype and trials effects on spontaneous exploration in the habituation/dishabituation test.**

| | Experiment | statistical test | Group (Main effects) | Trial (Main effects) | Trial x Genotype interactions (interaction) |
|---|---|---|---|---|---|
| **Fig. 9A** | **SOM** | | | | |
| | Global exploration (H1 to C3) | two-way r-m ANOVA | F(1,12)=1.67, P=0.22 | F(6,72)=29.48, P<0.0001 | F(6,72)=0.62, P=0.71 |
| | H1 to H4 | two-way r-m ANOVA | F(1,12)=0.672, P=0.43 | F(3,36)=16.95, P<0.0001 | F(3,36)=0.174, P=0.91 |
| | H4 to C+1 | two-way r-m ANOVA | F(1,12)=0.80, P=0.78 | F(1,12)=10.292, P<0.01 | F(1,12)=0.120, P=0.735 |
| | H5 to C+3 | two-way r-m ANOVA | F(1,12)=3.747, P=0.077 | F(1,12)=68.25, P<0.0001 | F(1,12)=0.198, P=0.19 |
| **Fig. 9B** | **SSTR2** | | | | |
| | Global exploration (H1 to C3) | two-way r-m ANOVA | F(1,13)=2.03, P=0.18 | F(6,78)=5.17, P<0.001 | F(6,78)=1.046, P=0.40 |
| | H1 to H4 | two-way r-m ANOVA | F(1,13)=1.342, P=0.27 | F(3,39)=6.191, P<0.01 | F(3,39)=0.202, P=0.89 |
| | H4 to C+1 | two-way r-m ANOVA | F(1,13)=0.368, P=0.55 | F(1,13)=6.676, P<0.05 | F(1,13)=0.004, P=0.95 |
| | H5 to C+3 | two-way r-m ANOVA | F(1,13)=3.041, P=0.10 | F(1,13)=4.693, P<0.05 | F(1,13)=2.061, P=0.17 |
| **Fig. 9C** | **SSTR4** | | | | |
| | Global exploration (H1 to C3) | two-way r-m ANOVA | F(1,14)=2.505, P=0.14 | F(6,84)=33.764, P<0.0001 | F(6,84)=0.958, P=0.46 |
| | H1 to H4 | two-way r-m ANOVA | F(1,14)=2.480, P=0.14 | F(3,42)=38.661, P<0.0001 | F(3,42)=0.26, P=0.85 |
| | H4 to C+1 | two-way r-m ANOVA | F(1,14)=5.258, P<0.05 | F(1,14)=84.384, P<0.0001 | F(1,14)=1.168, P=0.30 |
| | H5 to C+3 | two-way r-m ANOVA | F(1,14)=063, P=0.80 | F(1,14)=40.171, P<0.0001 | F(1,14)=0.936 P=0.35 |

| *All WT comparison* | Cohort effect | Trial effect | Cohort x Trial interaction |
|---|---|---|---|
| WT exploration time H1 to C3 | F(2,20)=14.004 P=0.0002 | F(6,120)=33,377 P<0.0001 | F(12,120)=3.099, P=0.0008 |
| WT exploration time H1 to H4 | F(2,20)=12.408 P=0.0003 | F(3,60)=27.453 P<0.0001 | F(6,60)=5.56 P<0.0001 |
| WT exploration time H4 to C+1 | F(2,20)=7.119 P=0.0046 | F(1,20)=32.207 p<0.0001 | F(2,20)=4.255 P=0.0289 |
| *WT SOM vs WT SSTR2* | *F(1,13)=8.393 P=0.0125* | *F(1,13)=7.715 P=0.0157* | *F(1,13)=0.663 P=0.43* |
| *WT SSTR2 vs WT SSTR4* | *F(1,14)=12.662 P=0.0031* | *F(1,14)=38.210 P<0.0001* | *F(1,14)12.116 P=0.0037* |
| WT exploration time H5 to C+3 | F(2,20)=7.202 P=0.0044 | F(1,20)=44.45 p<0.0001 | F(2,20)=2.201 P=0.1368 |
| *WT SSTR2 vs WT SSTR4* | *F(1,14)=11.376 P=0.0046* | *F(1,14)=16.830) P=0.0011* | *F(1,14)=1.985 P=0.1807* |

**Figure 10** **Statistical analysis of the olfactometer behavioral studies of SOM cohort (7 WT, 7 SOM KO)**

| | | | | Main effects | |
|---|---|---|---|---|---|
| | | **Parameters** | **Statistical test** | **Group** | **Session** |
| **Fig. 10A** | **Task1 learning** | **5 block-mean performance** | two-way r-m ANOVA | *F(1,11)=1.353 P=0.269 | F(6,66)=24.081 P<0.0001 |
| | **Task1 learning** | **Mean of the last three blocks** | one-way ANOVA Bonferroni corrected | F(1,12)=0.018 P=0.894 | |
| **Fig. 10B** | **Task1 learning** | **Blocks to Criterion** | one-way ANOVA Bonferroni corrected | F(1,12)=2.185 P=0.165 | |
| **Fig. 10C** | **Task1** | **Memory (21d)** | one-way ANOVA Bonferroni corrected | F(1,12)=0.064 P=0.805 | |
| | ***Task2 learning*** | **Mean of the last three blocks** | one-way ANOVA Bonferroni corrected | F(1,12)=4.038 P=0.068 | |
| **Fig.10D2** | **Detection task** | **Mean performance (10 blocks, all concentratior** | three-way r-m ANOVA | F(1,12)=1.538 P=0.239 | F(4,48)=9.130 P<0.0001 |
| | | | | | |
| | | | | | |
| | | | | | |
| | | **Mean performance (10 blocks, 1%)** | two-way r-m ANOVA | F(1,12)=0.255 P=0.623 | F(9,108)=16.009 P<0.0001 |
| | | **Mean performance (10 blocks, 0.1%)** | two-way r-m ANOVA | F(1,12)=0.826 P=0.381 | F(9,108)=2.305 P=0.021 |
| | | **Mean performance (10 blocks, 0.01%)** | two-way r-m ANOVA | F(1,12)=1.55 P=0.237 | F(9,108)=4.225 P<0.0001 |
| | | **Mean performance (10 blocks, 0.001%)** | two-way r-m ANOVA | F(1,12)=3.347 P=0.092 | F(9,108)=3.658 P=0.0005 |
| | | **Mean performance (10 blocks, 0.0001%)** | two-way r-m ANOVA | F(1,12)=3.508 P=0.086 | F(9,108)=1.699 P=0.098 |
| **Fig. 10E2** | **Detection task** | **Mean of the last three blocks (all concentration** | two-way r-m ANOVA | F(1,12)=4.899 P=0.047 | F(4,48)=52.637 P<0.0001 |
| | ***Task3 learning*** | **Mean of the last three blocks** | one-way ANOVA Bonferroni corrected | F(1,12)=0.054 P=0.821 | |
| **Fig. 10F2** | **Discrimination task** | **Mean performance (10 blocks, all mixtures)** | three-way r-m ANOVA | F(1,12)=0.005 P=0.945 | F(3,36)=30.926 P<0.0001 |
| | | | | | |
| | | | | | |
| | | | | | |
| **Fig. 10G2** | **Discrimination task** | **Mean of the last three blocks (all mixtures)** | two-way r-m ANOVA | F(1,12)=0.001 P=0.970 | F(3,36)=29.018 P<0.0001 |

***1 WT was not included (performing above 85% , blocks #29 and #30 missing)**

| | |
|---|---|
| | |
| **Trial** | **interaction** |
| | Group x Session F(6,66)=1.222 P=0.306 |
| | |
| | |
| | |
| | |
| F(9,108)=11.601 P<0.0001 | Group x Session F(4,48)=4.023 P=0.0068 |
| | Group x trial F(9,108)=1.227 P=0.286 |
| | Trial x Session F(36,432)=4.548 P<0.0001 |
| | Group x Session xTrial F(36,432)=0.771 P=0.829 |
| F(9,108)=0.99 P=0.453 | |
| F(9,108)=1.168 P=0.323 | |
| F(9,108)=1.125 P=0.035 | |
| F(9,108)=0.469 P=0.893 | |
| F(9,108)=0.632 P=0.768 | |
| F(4,48)=2.033 P=0.105 | |
| | |
| F(9,108)=3.55 P=0.0007 | Group x Session F(3,36)=0.149 P=0.929 |
| | Group x trial F(9,108)=0.499 P=0.872 |
| | Session xTrial F(27,324)=2.981 P<0.0001 |
| | Group x Session xTrial F(27,324)=0.614 P=0.936 |
| F(3,36)=0.088 P=0.966 | |

**Figure 10** **Statistical analysis of the olfactometer behavioral studies of SSTR2 cohort (8 WT, 7 SSTR2 KO)**

| | | | | Main effects | | |
|---|---|---|---|---|---|---|
| | | **Parameters** | **Statistical test** | **Group** | **Session** | **Trial** |
| | | | | | | |
| **Fig. 10A** | **Task1 learning** | **5 block-mean performance** | two-way r-m ANOVA | F(1,13)=0.717 P=0.412 | F(5,65)=60.059 P<0.0001 | |
| | ***Task1 learning*** | **Mean of the last three blocks** | one-way ANOVA Bonferroni corrected | F(1,13)=0.273 P=0.609 | | |
| **Fig. 10B** | **Task1 learning** | **Blocks to Criterion** | one-way ANOVA Bonferroni corrected | F(1,13)=0.521 P=0.483 | | |
| **Fig. 10C** | **Task1** | **Memory (21d)** | one-way ANOVA Bonferroni corrected | F(1,13)=1.136 P=0.306 | | |
| | ***Task2 learning*** | **Mean of the last three blocks** | one-way ANOVA Bonferroni corrected | F(1,13)=0.531 P=0.134 | | |
| **Fig.10D2** | **Detection task** | **Mean performance (10 blocks, all concentrations)** | three-way r-m ANOVA | F(1,13)=4.576 P=0.052 | F(4,52)=22.393 P<0.0001 | F(9,117)=9.860 P<0.0001 |
| | | | | | | |
| | | | | | | |
| | | | | | | |
| | | **Mean performance (10 blocks, 1%)** | two-way r-m ANOVA | F(1,13)=1.948 P=0.186 | F(9,117)=2.364 P=0.017 | |
| | | **Mean performance (10 blocks, 0.1%)** | two-way r-m ANOVA | F(1,13)=5.229 P=0.039 | F(9,117)=7.597 P<0.0001 | |
| | | **Mean performance (10 blocks, 0.01%)** | two-way r-m ANOVA | F(1,13)=5.151 P=0.041 | F(9,117)=9.53 P<0.0001 | |
| | | **Mean performance (10 blocks, 0.001%)** | two-way r-m ANOVA | F(1,13)=5.677 P=0.033 | F(9,117)=19.366 P<0.0001 | |
| | | **Mean performance (10 blocks, 0.0001%)** | two-way r-m ANOVA | F(1,13)=0.792 P=0.389 | F(9,117)=11.33 P<0.0001 | |
| **Fig. 10E2** | **Detection task** | **Mean of the last three blocks (all concentrations)** | two-way r-m ANOVA | F(1,13)=4.857 P=0.046 | F(2,26)=11.334 P<0.0001 | |
| | | **Mean of the last three blocks (1%)** | one-way ANOVA, Bonferroni corrected | F(1,13)=0.531 P=0.479 | | |
| | | **Mean of the last three blocks (0.1%)** | one-way ANOVA, Bonferroni corrected | F(1,13)=9.624 P=0.008 | | |
| | | **Mean of the last three blocks (0.01%)** | one-way ANOVA, Bonferroni corrected | F(1,13)=5.95 P=0.030 | | |
| | | **Mean of the last three blocks (0.001%)** | one-way ANOVA, Bonferroni corrected | F(1,13)=11.407 P=0.005 | | |
| | | **Mean of the last three blocks (0.0001%)** | one-way ANOVA, Bonferroni corrected | F(1,13)=0.064 P=0.804 | | |
| | ***Task3 learning*** | **Mean of the last three blocks** | one-way ANOVA Bonferroni corrected | F(1,13)=2.967 P=0.109 | | |
| **Fig. 10F2** | **Discrimination task** | **Mean performance (10 blocks, all mixtures)** | three-way r-m ANOVA | F(1,13)=5.203 P=0.040 | F(3,39)=66.524 P<0.0001 | F(9,117)=19.532 P<0.0001 |
| | | | | | | |
| | | | | | | |
| | | | | | | |
| | | **Mean performance (10 blocks, 100/0)** | two-way r-m ANOVA | F(1,13)=1.112 P=0.311 | F(9,117)=4.024 P<0.0001 | |
| | | **Mean performance (10 blocks, 80/20)** | two-way r-m ANOVA | F(1,13)=5.513 P=0.035 | F(9,117)=12.324 P<0.0001 | |
| | | **Mean performance (10 blocks, 60/40)** | two-way r-m ANOVA | F(1,13)=5.261 P=0.039 | F(9,117)=3.456 P=0.0008 | |
| | | **Mean performance (10 blocks, 55/45)** | two-way r-m ANOVA | F(1,13)=0.926 P=0.354 | F(9,117)=3.234 P=0.0015 | |
| **Fig. 10G2** | **Discrimination task** | **Mean of the last three blocks (all mixtures)** | two-way r-m ANOVA | F(1,13)=9.677 P=0.008 | F(3,39)=32.266 P<0.0001 | |
| | | | | | | |
| | | **Mean of the last three blocks (100/0)** | one-way ANOVA, Bonferroni corrected | F(1,13)=2.967 P=0.109 | | |
| | | **Mean of the last three blocks (80/20)** | one-way ANOVA, Bonferroni corrected | F(1,13)=7.124 P=0.019 | | |
| | | **Mean of the last three blocks (60/40)** | one-way ANOVA, Bonferroni corrected | F(1,13)=5.248 P=0.039 | | |
| | | **Mean of the last three blocks (55/45)** | one-way ANOVA, Bonferroni corrected | F(1,13)=1.412 P=0.256 | | |

| |
|---|
| **interaction** |
| |
| Group x Session F(5,65)=1.220 P=0.310 |
| |
| |
| |
| |
| Group x Session F(4,52)=1.462 P=0.227 |
| Group x Trial F(9,117)=1.487 P=0.161 |
| Trial x Session F(36,468)=9.639 P<0.0001 |
| Group x Session x Trial F(36.468)=1.640 P=0.013 |
| Group x Session F(9,117)=0.419 P=0.923 |
| Group x Session F(9,117)=0.752 P=0.661 |
| Group x Session F(9,117)=2.95 P=0.003 |
| Group x Session F(9,117)=2.493 P=0.012 |
| Group x Session F(9,117)=1.638 P=0.112 |
| Group x Session F(2,26)=2.898 P=0.031 |
| |
| |
| |
| |
| |
| |
| Group x Session F(3,39)=0.993 P=0.406 |
| Group x trial F(9,117)=2.099 P=0.035 |
| Trial x Session F(27,351)=1.789 P=0.010 |
| Group x Session x Trial F(27,351)=0.065 P=0.915 |
| Group x Session F(9,117)=1.028 P=0.422 |
| Group x Session F(9,117)=0.967 P=0.471 |
| Group x SessionF(9,117)=0.865 P=0.559 |
| Group x Session F(9,117)=1.204 P=0.299 |
| Group x Session F(3,39)=0.594 P=0.628 |
| |
| |
| |
| |
| |

**Figure 10** **Statistical analysis of the olfactometer behavioral studies of SSTR4 cohort (7 WT, 7 SSTR4 KO)**

| | | | | Main effects | |
|---|---|---|---|---|---|
| | | **Parameters** | **Statistical test** | **Group** | **Session** |
| | **Task1 learning** | **5 block-mean performance** | two-way r-m ANOVA | F(1,12)=2.163 P=0.167 | F(9,108)=17.293 P<0.0001 |
| | **Task1 learning** | **Mean of the last three blocks** | one-way ANOVA Bonferroni corrected | F(1,12)=1.036 P=0.329 | |
| **Fig. 10B** | **Task1 learning** | **Blocks to Criterion** | one-way ANOVA Bonferroni corrected | F(1,12)=1.840 P=0.199 | |
| **Fig. 10C** | **Task1** | **Memory (21d)** | one-way ANOVA Bonferroni corrected | F(1,12)=0.244 P=0.630 | |
| | ***Task2 learning*** | **Mean of the last three blocks** | one-way ANOVA Bonferroni corrected | F(1,12)=0.187 P=0.674 | |
| | **Detection task** | **Mean performance (10 blocks, all concentrations)** | three-way r-m ANOVA | F(1,12)=0.0003 P=0.986 | F(3,36)=58.515 P<0.0001 |
| | | | | | |
| | | | | | |
| | | | | | |
| **Fig. 10E3** | **Detection task** | **Mean of the last three blocks (all concentrations)** | two-way r-m ANOVA | F(1,12)=0.009 P=0.926 | F(3,36)=60.476 P<0.0001 |
| | ***Task3 learning*** | **Mean of the last three blocks** | one-way ANOVA Bonferroni corrected | F(1,12)=0.927 P=0.355 | |
| | **Discrimination task** | **Mean performance (10 blocks, all mixtures)** | three-way r-m ANOVA | F(1,12)=0.320 P=0.581 | F(3,36)=40.28 P<0.0001 |
| | | | | | |
| | | | | | |
| | | | | | |
| **Fig. 10G3** | **Discrimination task** | **Mean of the last three blocks (all mixtures)** | two-way r-m ANOVA | F(1,12)=0.041 P=0.843 | F(3,36)=22.66 P<0.0001 |

| | **Interaction** |
|---|---|
| **Trial** | **Group x Session  (unless specified)** |
| | F(9,108)=0.775 P=0.639 |
| | |
| | |
| | |
| | |
| F(9,108)=5.875 P<0.0001 | Group x Session F(3,36)=0.281 P=0.839 |
| | Group x trial F(9,108)=0.429 P=0.917 |
| | Trial x Session F(27,324)=1.376 P=0.105 |
| | Group x Session x Trial F(27,234)=1.372 P=0.107 |
| | Group x Session F(3,36)=1.121 P=0.353 |
| | |
| F(9,108)=30.888 P<0.0001 | Group x Session F(3,36)=0.164 P=0.920 |
| | Group x trial F(9,108)=1.331 P=229 |
| | Trial x Session F(27,324)=2.554 P<0.0001 |
| | Group x Session x Trial F(27,234)=0.806 P=0.744 |
| | Group x SessionF(3,36)=0.401 P=0.753 |

**Statistical analysis of the olfactometer behavioral studies**

| ***All WT*** | test |
|---|---|
| **Performance accuracy after Anisole/Cineole learning** | one-way ANOVA (Bonferroni correction) SOM vs SSTR2 |
| **Blocks to criterion Task1** | one-way ANOVA (Bonferroni correction) SOM vs SSTR2 vs SSTR4 |
| **Performance accuracy after Task2 learning** | one-way ANOVA (Bonferroni correction) SOM vs SSTR2 vs SSTR4 |
| **Detection test: 10 blocks, all concentrations** | three-way r-m ANOVA SOM vs SSTR2 |
| **Detection test: last 3 blocks, all concentrations** | three-way r-m ANOVA SOM vs SSTR2 |
| **Performance accuracy after Task3 learning** | one-way ANOVA (Bonferroni correction) SOM vs SSTR2 vs SSTR4 |
| **Discrimination test: 10 blocks, all mixtures** | three-way r-m ANOVA SOM vs SSTR2 vs SSTR4 |
| **Discrimination test: last 3 blocks, all mixtures** | three-way r-m ANOVA SOM vs SSTR2 vs SSTR4 |
| | |

| Cohort effect | Session effect | Cohort x session interaction |
|---|---|---|
| F(1,13)=0.124 P=0.729 | | |
| F(2,19)=9.373 P=0.002 (SOM vs SSTR2 ns, SOM vs SSTR4 P=0.0001 SSTR2 vs SSTR4 P<0.0001, Bonferroni corr.) | | |
| F(2,19)=0.482 P=0.621 | | |
| F(1,13)=0.125 P=0.729 | F(4,52)=3.87 P=0.008 | F(4,52)=26.068 P<0.0001 |
| F(1,13)=0.796 P=0.389 | F(4,52)=47.292 P<0.0001 | F(4,52)=0.750 P=0.6119 |
| F(2,19)=1.846 P=0.185 | | |
| F(2,19)=1.063 P=0.3651 | F(3,57)=83.065 P<0.0001 | F(6,57)=0.750 P=0.612 |
| F(2,19)=3.027 P=0.072 | F(3,57)=47.176 P<0.0001 | F(6,57)=0.357 P=0.903 |
| | | |
